# Synaptic loss in alcohol use disorder: clinical and mechanistic insights from a PET imaging study

**DOI:** 10.1172/JCI204460

**Published:** 2026-04-01

**Authors:** Sarah K. Royse, Rajesh Narendran

**Affiliations:** 1 Departments of Radiology and; 2 Department of Psychiatry, University of Pittsburgh, Pittsburgh, Pennsylvania, USA.

## Abstract

Alcohol use disorder (AUD) is linked with changes in brain structure and function, with robust evidence for neurodegenerative changes, including synaptic loss in preclinical models. Developing therapeutic strategies to target synaptic loss will require human studies that clarify their clinical relevance of these changes. In the current issue, Zakiniaeiz et al. demonstrate that AUD and alcohol consumption are associated with lower synaptic vesicle glycoprotein 2a (SV2A) expression, indexed by regional [^11^C]UCB-J PET. This is, to our knowledge, the first in vivo evidence of relationships between synaptic density and alcohol use, and, as such, it represents an important step toward understanding how AUD influences brain structure and function. Here, we describe two longstanding clinical issues in the AUD population — relapse and dementia risk — and how the results of the present study may guide future investigations of these issues.

Alcohol use is strongly associated with neurodegeneration and alterations in neuromorphology and neuroplasticity in preclinical models (1). MRI studies in humans corroborate these findings at a macrostructural level (2). While restoring synaptic loss may be an effective therapeutic strategy for preventing relapse or neurocognitive sequelae in alcohol use disorder (AUD), more comprehensive evidence for its clinical relevance in humans is needed. In this issue, Zakiniaeiz et al. (3) leveraged advances in PET imaging to quantify synaptic density using the synaptic vesicle glycoprotein 2A (SV2A) tracer [11C]UCB-J. In a comparison of 32 people with AUD with healthy control (HC) participants, they reported deficits in synaptic density that increased alongside drinking severity (Figure 1). Below, we will describe the strengths of this study’s design and discuss the interpretation and implications of these findings.

## Study strengths and interpretation

In this issue of the *JCI*, Zakiniaeiz et al. (3) showed that (a) SV2A, quantified as [^11^C]UCB-J PET binding potential (BP_ND_), was 11% lower in the frontal cortex, hippocampus, striatum, and cerebellum in individuals with AUD compared with HC participants; (b) in those with AUD, more drinks per day was associated with lower [^11^C]UCB-J BP_ND_ in the frontal cortex and striatum; and (c) performance on an executive function task was worse for individuals with AUD than HCs.

To understand the relevance of results, it is necessary to appreciate the many strengths of this study. (a) A newly developed, robust radiotracer, [^11^C]UCB-J, was used in a relatively large number of individuals with AUD by mechanistic PET investigation standards; (b) the study included a large proportion of participants with mild-to-moderate AUD severity and minimal-to-negligible psychiatric, substance use, and medical comorbidities; (c) few individuals were on neurochemistry-altering psychotropic medications; (d) AUD and HC participants were matched for age, sex, tobacco use, and cannabis use; (e) overnight abstinence from alcohol was verified, and the absence of substantial alcohol withdrawal symptoms was documented prior to PET, ultimately confirming that there were no acute effects of alcohol intoxication or withdrawal on [^11^C]UCB-J BP_ND_; (f) an arterial input function was used to demonstrate a lack of significant between-group differences in nonspecific binding (V_ND_) in the reference region; (g) analyses used a partial volume correction to account for between-group differences in gray matter volume; and (viii) rigorous statistical methods were implemented.

There are also interpretive challenges to consider. [^11^C]UCB-J binds to SV2A, which is present in all presynaptic vesicles of neurons, irrespective of neurotransmitter class, given its role in regulating the readily releasable pool of synaptic vesicles containing GABA and glutamate (4, 5). Primate and human postmortem studies report a linear correlation between SV2A and synaptophysin, the gold-standard IHC marker of synaptic density (6, 7). It is therefore reasonable to assume, as Zakiniaeiz et al. did, that lower [^11^C]UCB-J BP_ND_ in individuals with AUD relative to HC participants reflects lower synaptic density. However, alternative in terpretations deserve consideration. Given the abundance of glutamatergic and GABAergic neurons in the brain, a large fraction of [^11^C]UCB-J binding likely represents SV2A in these excitatory and inhibitory synapses (4). Because repeated alcohol use alters GABA-ergic and glutamatergic transmission (8), it is possible that lower SV2A in people with AUD reflects a compensatory adaptation to these chronic neurochemical changes. In other words, compared with HCs, those with AUD may exhibit a reduction in SV2A proteins (or vesicles in which they are present) without a corresponding decrease in synaptic density. Such caution is further warranted because individuals with AUD were scanned after abstaining for 2 ± 2 days, as opposed to typical AUD PET studies that assess abstinence lasting for weeks, with the aim of documenting more stable changes. Additionally, postmortem studies in humans have reported a loss of both neurons and synapses in brain regions of heavy drinkers compared with controls (9–11). Thus, it is possible that lower SV2A in individuals with AUD in the PET study reflects chronic alcohol consumption–induced neurodegeneration, which would also be associated with fewer synapses. Regardless of whether lower [^11^C]UCB-J BP_ND_ in AUD indicates a reversible or irreversible change — due to fewer SV2A proteins, vesicles, synapses, neurons, or a combination — it is an important result.

It is also worth mentioning the nonsignificant, approximately 8% lower SV2A binding (V_T_) in AUD versus HC participants in the centrum semiovale, a subcortical white matter area that was used as the reference region. As the authors noted, this binding difference in the reference region may underestimate the magnitude of group differences between regions, ultimately supporting the strength of their findings. Still, this group difference deserves careful consideration. Previous evidence suggests that there may be some, albeit small, true specific binding of [^11^C]UCB-J in white matter (12). Thus, the group difference in centrum semiovale V_T_ may reflect poorer white matter integrity in the participants with AUD versus HCs. This interpretation is consistent with work showing that people with AUD show widespread white matter alterations (13).

The sum of this work represents an important step toward better understanding the influence of AUD on brain structure and function. Replicating and building upon this study will be critical to the field and may ultimately help answer longstanding clinical questions.

## Do lower SV2A levels promote neurochemical changes and relapse?

Alcohol withdrawal, characterized by predominantly physical symptoms during early abstinence and psychological symptoms in prolonged abstinence, drives negative reinforcement and contributes to relapse. Relapse may be driven partially by chronic neurochemical adaptations during withdrawal and abstinence, including both inhibitory GABA-ergic and excitatory glutamatergic systems (8). Current treatment protocols include using GABA-ergic medications (e.g., benzodiazepines) during acute withdrawal and the glutamate modulator acamprosate (or the μ-opioid antagonist naltrexone) to promote abstinence. No FDA-approved medication simultaneously targets inhibitory and excitatory neurotransmission to prevent relapse. On the basis of SV2A-knockout mouse studies (14), it is tempting to speculate that lower SV2A expression in AUD preferentially diminishes GABA-ergic over glutamatergic transmission, thereby altering the brain’s inhibitory-excitatory balance, contributing to withdrawal symptoms, negative emotions, and relapse to alcohol (15). Additional studies of [^11^C]UCB-J that link lower expression levels of SV2A to relapse in AUD will be necessary to clarify its clinical relevance, as will studies that examine SV2A-upregulating medications (such as psychedelics) in preventing relapse, as discussed by the authors.

## Does alcohol-induced synaptic loss predict a future dementia diagnosis?

By 2030, the older adult population (aged 65 years and older) will be more than double that of 2000, an increase driven by the Baby Boomer generation. Baby Boomers consume more alcohol than previous generations (16), which will likely contribute to an increase in dementia incidence and prevalence. Indeed, the hazard for developing all-cause dementia is two-fold higher among individuals with AUD/heavy drinkers than the general population (17–19). Mendelian randomization approaches also suggest that any alcohol consumption increases the dementia risk (20). Despite strong evidence of an association between heavy drinking and dementia, translational studies focused on mechanisms such as Aβ aggregates, hyperphosphorylated tau, or α-synuclein have yielded negative results (21–24). Thus, there is a pressing need to characterize this relationship, particularly by identifying AUD-associated neuropathological insults beyond established dementia causes.

One promising mechanism supported by postmortem data is the loss of synapses and neurons in AUD (9–11). MRI studies have shown widespread regional reductions in gray matter volumes (GMVs) in people with AUD versus HCs (2). In middle-aged/older individuals with AUD, lower GMV is associated with worse neurocognitive impairments in attention and executive function (24, 25). However, MRI studies are limited, as they cannot distinguish if lower GMV indicates loss of neurons, synapses (or both), glial cells, or a reversible shrinkage of tissue volume. The findings of Zakiniaeiz et al. provide the first evidence, to our knowledge, of an alcohol dose–dependent loss of synapses (and possibly neurons) in individuals with AUD. This loss has the potential to increase the risk of developing dementia, perhaps by lowering the brain reserve, making the brain more susceptible to pathological insult conferred by characteristic dementia-causing pathology. Longitudinal studies are necessary, as these can determine if relatively lower SV2A expression in individuals with AUD versus HCs persists over time and, ultimately, if this change can be attributed to synaptic loss.

Linking lower SV2A expression to major neurocognitive impairments in middle-aged and older adults with AUD is crucial. Notably, Zakiniaeiz et al. did not detect (a) differences in verbal learning or memory performance between AUD and HC participants or (b) a relationship between [^11^C]UCB-J and cognitive performance. This may be because they did not perform a formal neurocognitive battery, which would be more sensitive in detecting subtle differences and associations. The lack of difference in memory performance is also not surprising, the participants with AUD were young (43 ± 13 years of age). As such, studies that build upon this work by using more exhaustive neuropsychological assessments in middle-aged/older people with AUD will provide valuable information surrounding this relationship.

The present work also provides a strong basis for investigating both microglial activation and synaptic loss as plausible mechanisms contributing to dementia in people with a history of chronic heavy drinking. As discussed by Zakiniaeiz et al., the role of microglia in regulating synaptic density in chronic alcohol use may hold the key to preventing synaptic loss and neurocognitive deficits. Rodent studies show that chronic alcohol exposure increases microglial activation and expression of TREM2, a protein that signals microglial responses to neurodegeneration (26). These studies have also found that knocking out TREM2 in hippocampal microglia increases dendritic spine density, decreases synaptic protein loss, and reduces memory loss following chronic alcohol exposure. Furthermore, chronic binge drinking in mice increases microglial engulfment capacity, promotes aberrant synaptic pruning, and results in a loss of excitatory synapses in the prefrontal cortex (27). These basic investigations linking TREM2-mediated microglial function to glutamatergic synaptic loss, which can impair learning and memory, may explain a future dementia diagnosis for individuals with a history of chronic heavy drinking.

## Conclusion

In summary, the lower SV2A levels in individuals with heavy-drinking AUD reported by Zakiniaeiz et al. is a significant discovery that warrants further investigation. Understanding this finding is critical to addressing some of the clinical questions that have long plagued AUD research, for example, how to develop medications that can target both inhibitory and excitatory transmission to promote abstinence, and how drinking increases the risk of Alzheimer’s disease without altering Aβ or hyperphosphorylated tau accumulation.

## Funding support

This work is the result of NIH funding, in whole or in part, and is subject to the NIH Public Access Policy. Through acceptance of this federal funding, the NIH has been given a right to make the work publicly available in PubMed Central.

- NIH grant RF1AG096215 (SKR).

## Figures and Tables

**Figure 1:**
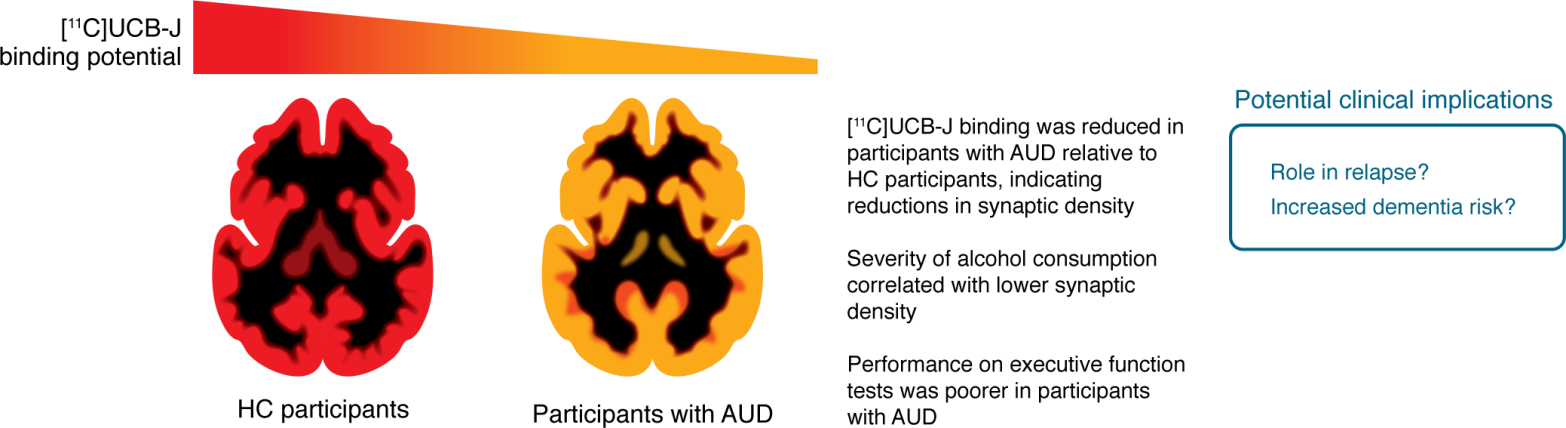
Alcohol use disorder is associated with reductions in PET-quantified synaptic density. Zakiniaeiz et al. (3) used PET imaging of the SV2A tracer [11C]UCB-J to determine that synaptic density was reduced in participants with AUD compared with HCs. Moreover, the severity of alcohol consumption among the participants with AUD correlated with lower synaptic density. These findings raise further questions about the relationship of changes in synaptic density to the risk of relapse and dementia in people with AUD.

## Version 1 04/01/2026

Electronic publication
